# Inflammatory, oxidative, and neurotrophic profiles in monozygotic twins discordant for pain-related TMD

**DOI:** 10.1007/s11033-026-11902-y

**Published:** 2026-05-14

**Authors:** Laís Valencise Magri, Melissa Oliveira Melchior, Cecília Nogueira Tavares Peixeiro, Vitória Carolina Rondon-Pereira, Margarete Ribeiro-Dasilva, Kranya Victoria Díaz-Serrano, Riccardo Lacchini, Edilaine Cristina da Silva Gherardi-Donato, Christie Ramos Andrade Leite-Panissi

**Affiliations:** 1 Department of Restorative Dentistry, School of Dentistry of Ribeirao Preto, University of São Paulo, Ribeirão Preto, São Paulo, Brazil; 2 Department of Psychiatric Nursing, College of Nursing of Ribeirão Preto, University of São Paulo, Ribeirão Preto, São Paulo, Brazil; 3 PAHO/WHO Collaborating Centre for Nursing Research Development, Ribeirão Preto, São Paulo, Brazil; 4 Department DN-Prosthodontics Division, College of Dentistry, University of Florida, Gainville, FL USA; 5 Department of Pediatric Dentistry, Ribeirão Preto School of Dentistry, University of Sao Paulo, Ribeirão Preto, São Paulo, Brazil; 6 Department of Psychology, Faculty of Philosophy, Science and Letters of Ribeirão Preto, University of São Paulo, Ribeirão Preto, Brazil

**Keywords:** Temporomandibular disorders, Orofacial pain, Monozygotic twins, Inflammation, Oxidative stress, Neurotrophic factors, Biomarkers, Chronic pain

## Abstract

This study investigated the biological mechanisms underlying temporomandibular disorder (TMD) pain in monozygotic twins discordant for the condition, isolating environmental factors from genetic influence. Twenty women (ten pairs of discordant twins) underwent standardized examinations and venous plasma analysis. Inflammatory biomarkers (IL-6, IL-10), oxidative markers (MDA, SOD, catalase), matrix remodeling proteins (MMP-2, MMP-9, TIMP-1, TIMP-2), and neurotrophic factors (BDNF, β-NGF, α2M) were measured. Twins with painful TMD demonstrated greater pain severity, functional interference, and mechanical sensitivity compared to discordant controls. Significant within-pair differences were identified in IL-6, IL-6/IL-10 ratio, MDA/SOD ratio, MMP-9, TIMP-2, and BDNF levels. Pro-inflammatory and oxidative indices positively correlated with pain intensity and palpation sensitivity, while reduced BDNF associated with greater symptom burden. Principal component analysis revealed a dominant inflammatory-oxidative profile that discriminated painful twins from controls. In genetically identical individuals, painful TMD associates with selective peripheral biological alterations involving inflammation, oxidative imbalance, and extracellular matrix remodeling. These findings could demonstrate that environmental and experiential factors biologically may contribute to vulnerability to chronic orofacial pain, highlighting the importance of epigenetic mechanisms in TMD pathogenesis beyond genetic predisposition. This article identifies specific peripheral biomarker profiles distinguishing monozygotic twins with and without painful TMD, and could show that inflammation, oxidative imbalance, matrix remodeling, and reduced neurotrophic support characterize the painful phenotype. These mechanistic insights could enable clinicians to develop targeted, biologically informed therapeutic strategies and potentially predict pain vulnerability in genetically susceptible individuals.

## Introduction

Temporomandibular disorders (TMD) are among the most frequent chronic orofacial pain conditions and arise from the interaction of biological, emotional, and environmental influences [1–4]. Large cohort studies have shown that individuals with painful TMD often present heightened mechanical sensitivity and altered nociceptive modulation, indicating contributions from both peripheral and central pain mechanisms [1–3, 5]. Despite the advances in characterizing clinical phenotypes, the mechanisms underlying why some individuals develop persistent pain while others with comparable life experiences remain pain-free are not fully understood.

Psychological and behavioral factors appear to shape pain experience and disability in TMD [1, 2, 5, 6]. Patients frequently exhibit greater hyperalgesia, sleep disturbances, emotional distress, and attentional patterns directed toward pain, and these features are linked to more intense symptoms and functional compromise [1, 3, 5, 6]. Clinically, this includes increased muscle tenderness, familiar pain evoked during palpation, and pain with mandibular movement, supporting an interaction between somatic sensitivity and behavioral responses [1–3, 6]. These findings reinforce the importance of considering pain perception, behavior, and clinical presentation as interrelated domains in TMD.

Twin and family studies have consistently demonstrated that a proportion of TMD pain risk is attributable to inherited factors, supporting a heritable component to the disorder while also highlighting the importance of environmental and experiential influences [4, 7–9]. Monozygotic twins, in particular, provide a unique research model given their identical genetic profile, and discordance in pain presentation within twin pairs suggests that biological processes related to life experiences, stress exposure, and behavioral adaptation may contribute meaningfully to disease expression [1, 5, 7, 8].

Current research has increasingly focused on biological systems associated with pain persistence, including immune mediators, oxidative processes, and neurobiological adaptations [1, 3, 6, 10]. Epigenetic mechanisms such as DNA methylation and chromatin modifications have been shown to influence gene expression relevant to nociception and inflammatory signaling [2, 3, 10, 11]. Biomarker-based investigations have identified inflammatory cytokines, oxidative stress indicators, and matrix-regulating molecules associated with TMD [12–15], although evidence remains heterogeneous and few studies have adopted genetically controlled approaches [12, 14–16].

Despite important advances, the specific biological characteristics associated with painful TMD in individuals who share identical genetic backgrounds are still unclear. Our previous research with monozygotic twins discordant for painful TMD demonstrated differences in clinical pain responses and behavioral features even in the absence of genetic variability [2, 5–7]. Building on these findings, the present study investigates whether peripheral inflammatory markers, oxidative stress indicators, and neurotrophic factors differ between genetically identical women discordant for painful TMD. We hypothesize that twins with painful TMD will show higher inflammatory activity and altered biomarker patterns relative to their pain-free co-twins. Therefore, the objective of this study was to characterize biological signatures associated with painful TMD in monozygotic twins and relate these molecular findings to clinical pain expression.

## Methods

### Study design and ethical approval

This investigation employed a cross-sectional, discordant monozygotic twin design to examine biological and clinical correlates of painful TMD. By comparing genetically identical individuals who differ in pain status, this design minimizes confounding from inherited susceptibility, population stratification, and shared early-life environment, thereby strengthening inferences regarding environmentally shaped biological variation. Although cross-sectional in nature and therefore not suited for causal inference, the discordant twin approach enhances internal validity by isolating phenotypic divergence that arises after the period of shared genetic and early developmental influences.

Monozygotic status was confirmed through genetic testing using blood samples, following standard procedures for zygosity determination in twin research. This method is considered the gold standard and ensures high accuracy in distinguishing monozygotic from dizygotic twins.The present study expands upon a previously published analysis in the same cohort that characterized cognitive-emotional and somatosensory features of the painful phenotype [5].

The study protocol was approved by the Research Ethics Committee of Faculty of Philosophy, Sciences and Letters of Ribeirãão Preto,University of Sãão Paulo (CAAE: 98129918.6.0000.5407), which includes two representatives of patients who are users of the dental services provided by this institution. Participants were recruited voluntarily and provided written informed consent before enrollment. Confidentiality, autonomy, and the right to withdraw were fully guaranteed throughout the study.

### Participants

A total of 20 female monozygotic twins (10 discordant pairs) aged 18 to 55 years participated in the study. Participants were recruited via institutional databases, social media, and community announcements as described in the original study [5]. Monozygotic status was confirmed through assessment of phenotypic similarity assessment and parental confirmation, following widely accepted protocols for twin-research.

### Diagnosis of painful TMD

The diagnosis of painful TMD was established using the validated Brazilian Portuguese version of the Diagnostic Criteria for Temporomandibular Disorders (DC/TMD)[17], administered by clinicians formally trained and calibrated in DC/TMD methodology. The clinical examination followed the standardized Axis I protocol and included systematic palpation of the masticatory muscles and TMJ, assessment of mandibular range of motion, and evaluation of pain provocation and reproduction of familiar symptoms. This structured approach ensured diagnostic reliability and minimized examiner-related variability, an important consideration in twin studies where misclassification could obscure true within-pair differences.

### Inclusion criteria

Participants were eligible if they met all of the following criteria: female monozygotic twin, aged 18 to 55 years; one twin reporting ≥ 3 months of orofacial pain and fulfilling DC/TMD diagnostic criteria for painful TMD (myalgia and/or arthralgia) and the co-twin presenting *no* DC/TMD diagnosis and *no* persistent orofacial pain. This definition ensured clear discordance while allowing for the possibility of isolated or subclinical symptoms in the control twin, provided they did not satisfy diagnostic thresholds.

### Exclusion criteria

Exclusion criteria were defined to avoid confounding from concurrent conditions or treatments known to influence pain or inflammatory pathways. Twins were excluded if they were undergoing or had recently undergone TMD-related therapy (e.g., interocclusal appliances, physiotherapy, photobiomodulation/laser therapy, acupuncture), or were using analgesics, anti-inflammatories, or centrally acting medications. Additional exclusions included history of head or neck tumors, trauma, or surgery; diagnosed neurological disease; severe psychiatric disorders (except anxiety or depression, due to their high prevalence in chronic pain populations); and pregnancy or breastfeeding. These criteria minimized biological heterogeneity unrelated to TMD.

### Control of menstrual cycle phase

Because both TMD symptoms and inflammatory markers exhibit menstrual cycle–related variability [18, 19], all participants with regular cycles were assessed during the follicular phase. This phase was selected because estrogen rises gradually and progesterone remains low and stable, producing a more uniform endocrine environment. Conducting assessments during the follicular phase reduces hormonal fluctuations known to influence pain sensitivity, neuroimmune activity, and inflammatory mediator levels, thereby enhancing internal consistency across participants and reducing endocrine-related confounding.

### Clinical assessment

Clinical examinations were performed in accordance with DC/TMD Axis I guidelines, using standardized procedures for manual palpation of masticatory and cervical musculature and for the evaluation of mandibular functional movements [17]. Trained examiners applied controlled, criterion-based digital pressure to predefined anatomical sites to assess pain provocation, reproduction of familiar symptoms, and elicitation of referred pain patterns. These procedures followed established diagnostic thresholds for muscle tenderness and mechanical provocation of TMD-related symptoms, thereby minimizing examiner-related variability and enhancing diagnostic reliability within the twin pairs.

Mandibular movement assessment—comprising maximal unassisted and assisted opening, lateral excursions, and protrusion—was conducted to document movement-evoked pain and features of functional impairment consistent with painful myogenous TMD. Pain severity and functional interference were quantified using the Brief Pain Inventory (BPI), which offers validated, continuous indices of pain intensity and its impact on daily functioning, supporting comprehensive clinical characterization [20].

Objective measures of nociceptive sensitivity were incorporated through quantification of the proportion of palpation-positive sites relative to the total number of evaluated regions. This approach allowed systematic capture of localized muscle tenderness and mechanical hyperalgesia across standardized examination sites. In addition, pain elicited during mandibular movement was recorded, alongside differentiation among familiar pain on palpation, referred pain, and palpation-induced familiar headache. These distinctions reflect well-defined DC/TMD categories and permit more precise mapping of somatosensory responses relevant to the TMD pain phenotype.

Together, these multidimensional clinical measures—summarized in Table 1—capture a pattern of increased mechanical sensitivity, widespread palpation tenderness, movement-provoked pain, and functional limitation characteristic of painful myogenous TMD. This rigorously assessed clinical profile provides a robust foundation for integrating peripheral biomarker data and interpreting biological correlates of pain expression in a genetically controlled context.

### Biological sample collection and processing

Venous blood samples were collected in the morning following an overnight fast to minimize circadian influences and diet-related metabolic variability on inflammatory, oxidative, and neurotrophic biomarkers. Standardized phlebotomy procedures were used, and samples were processed immediately after collection to reduce pre-analytical degradation. Whole blood was centrifuged under controlled conditions, and the resulting plasma was aliquoted into sterile, coded polypropylene tubes to prevent repeated freeze–thaw cycles and preserve analyte integrity. Plasma samples were stored at − 80 °C until analysis, following established recommendations for long-term biomarker stability.

To minimize analytical bias, all laboratory personnel performing biomarker quantification were blinded to the clinical status of each twin. Blinding procedures ensured that sample handling, assay performance, and data recording were not influenced by knowledge of group assignment, thereby increasing the internal validity of the biomarker measurements.

### Biomarker analysis

Biomarkers were selected based on their mechanistic relevance in inflammation, oxidative stress, extracellular matrix regulation, and neuroplasticity, domains previously implicated in chronic musculoskeletal pain. Assays were performed following the manufacturers’ protocols.

Inflammatory mediators and matrix remodeling factors were quantified by sandwich-type ELISA (Enzyme-Linked Immunosorbent Assay) (R&D Systems, Minneapolis, MN, USA). Specifically, Quantikine kits were used for IL-6 (Catalog RDSY-D6050-96SW), IL-10 (Catalog RDSY-D1000B-96SW) and TNF-α (Catalog RDSY-DTA00D-9SW). For matrix remodeling, DuoSet kits were used for MMP-2 (Catalog RDSY-DY902-1KIT), MMP-9 (Catalog RDSY-DY911-1KIT), TIMP-1 (Catalog RDSY-DY970-1KIT), TIMP-2 (Catalog RDSY-DY971-1KIT), and α-2-macroglobulin (Catalog RDSY-DY1938-96SW).

Oxidative stress markers were analyzed by colorimetric/spectrophotometric assays (Cayman Chemical, Ann Arbor, MI, USA). Kits used were for Malondialdehyde (MDA) (TBARS Assay Kit, Catalog CAYM-10009055–96 W), Superoxide Dismutase (SOD) (Assay Kit, Catalog CAYM-706002–96 W), and Catalase (Assay Kit, Catalog CAYM-707002–96 W).

### Statistical analysis

Within-pair differences (Δ = TMD – Control) were calculated for all biomarkers and clinical variables to minimize genetic and shared early environmental confounding inherent to monozygotic discordant twin designs. Data distribution was examined using the Shapiro–Wilk test, and variables with strong skew or extreme values were inspected using Q–Q plots and leverage diagnostics. Descriptive statistics were reported as means and standard deviations for raw values and as medians and interquartile ranges when distributional asymmetry was present.

Inferential comparisons between affected and control twins were conducted using paired statistical tests. Paired t-tests were applied when Δ values met normality assumptions, whereas non-normally distributed variables or those with tied ranks were analyzed using Wilcoxon signed-rank tests. Correlations between Δ clinical measures (pain severity, functional interference, movement-evoked pain, and palpation outcomes) and Δ biomarker concentrations were assessed using Pearson coefficients (r), with significance defined as *P* < 0.05. Correlation matrices were visualized to identify clusters of clinically relevant associations.

To explore multivariate patterns, principal component analysis (PCA) was performed on z-standardized Δ biomarker values. Clinical variables were then projected as supplementary correlation vectors onto the PCA solution (PC1 and PC2) to evaluate their alignment with the dominant biological dimensions. Clinical vectors with |r| > 0.4 were interpreted as meaningful contributors to component structure. All analyses and visualizations—including paired-difference plots, correlation heatmaps, and PCA biplots—were conducted in R (version 4.4.0; R Foundation for Statistical Computing, Vienna, Austria).

## Results

### Clinical characteristics

As required by the discordant-twin design, in all ten pairs one twin fulfilled full DC/TMD criteria for painful TMD while the co-twin did not. Importantly, all control twins received the formal DC/TMD classification of *absence of TMD*; however, several exhibited subclinical findings, such as mild tenderness on isolated palpation sites or occasional movement-associated discomfort, without reaching diagnostic thresholds. This nuance helps contextualize the within-pair differences observed and clarifies that the control phenotype represents the *absence of a diagnosable disorder*, rather than a completely symptom-free state.

Group-level comparisons showed that painful twins reported higher mean pain levels (BPI-severity: 3.9 ± 1.5; BPI-interference: 3.4 ± 1.6), whereas their co-twins reported lower scores (2.5 ± 1.6 and 2.0 ± 1.7, respectively). Nevertheless, as shown in Table 1, a few control twins reported low-intensity pain or minimal interference, which produced negative Δ values in some pairs (e.g., pairs 1, 9, and 10). These cases illustrate the expected continuous distribution of pain experiences even in the absence of a formal diagnosis.

Similarly, across DC/TMD clinical examination domains—percentage of painful palpation points, movement-evoked pain, familiar pain, referred pain, and headache-related palpation pain—affected twins generally exhibited higher values. Still, control twins occasionally displayed isolated tenderness or temporal muscle sensitivity, again without meeting diagnostic criteria. Consequently, Δ values varied in magnitude and direction, ranging from small negative differences in some pairs to large positive contrasts in others. Overall, Table 1 demonstrates that although painful twins consistently showed greater clinical burden, the discordant-twin model naturally captures a graded, subclinical–to–clinical spectrum, reinforcing the biological relevance of examining within-pair differences beyond genetic susceptibility.

**Fig. 1:**
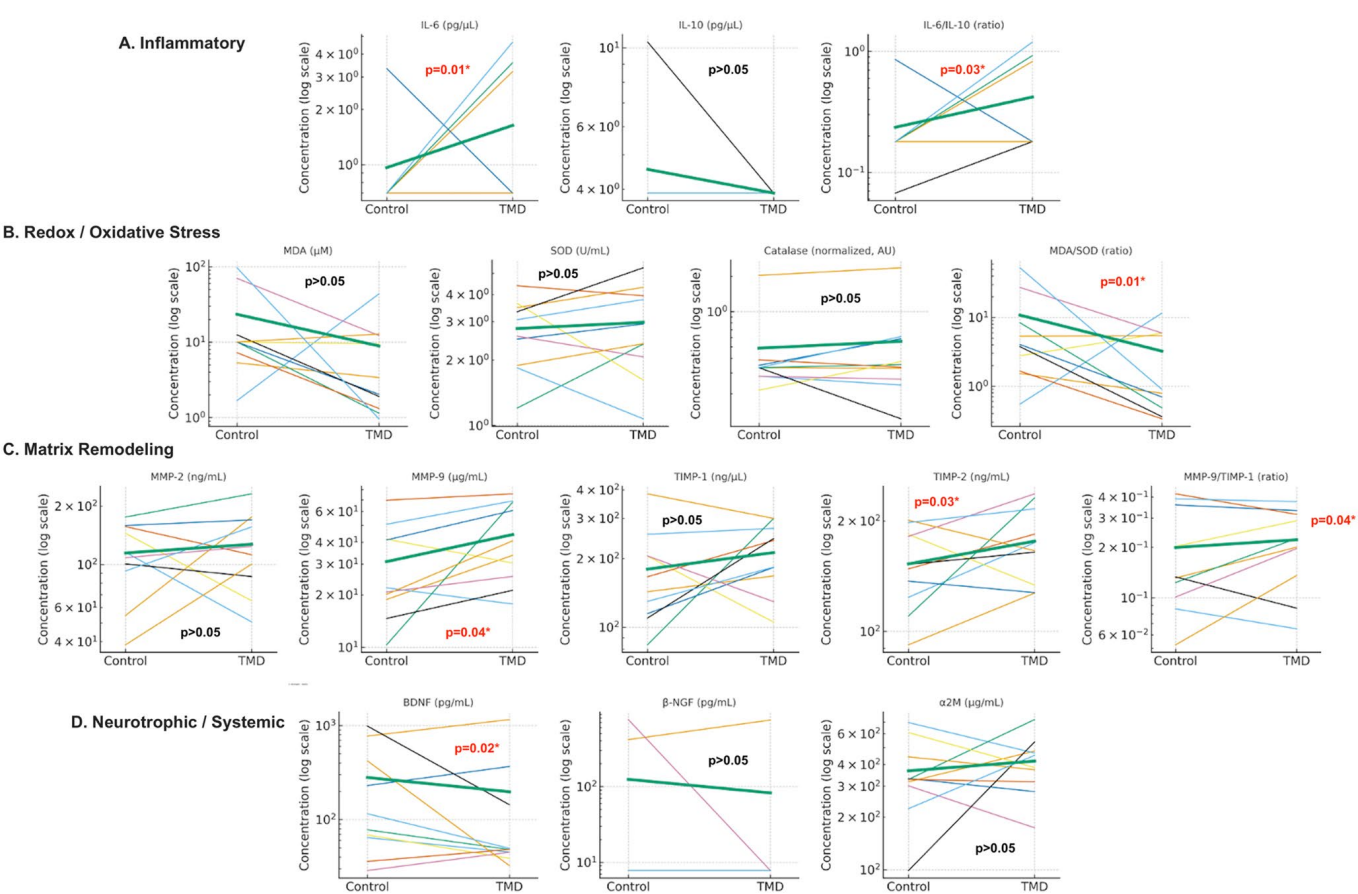
Each line connects values from the control twin (left) to her affected co-twin (right),illustrating the direction and magnitude of within-pair differences. Thinner lines representindividual twin pairs, whereas the thicker green line indicates the group mean. Biomarkers are grouped by biological domain: (**A**) Inflammatory — IL-6, IL-10, and IL-6/IL-10; (**B**) Redox/Oxidative Stress — MDA, SOD, CAT, and MDA/SOD; (**C**) Matrix Remodeling — MMP-2,MMP-9, TIMP-1, TIMP-2, and MMP-9/TIMP-1; and (**D**) Neurotrophic/Systemic Factors — BDNF,β-NGF, and α2M. Within-pair p-values from Wilcoxon tests are displayed in each panel;significant differences were observed for IL-6, IL-6/IL-10, MDA/SOD, MMP-9, TIMP-2, MMP-9/TIMP-1, and BDNF. Concentrations are shown on a logarithmic scale when appropriate toaccommodate biomarker variability

Figure 1 displays within-pair differences in biomarker concentrations between monozygotictwins discordant for painful TMD, revealing a pattern of selective rather than globalbiological divergence. Significant increases were observed in affected twins for IL-6, IL-6/IL-10, MDA/SOD, MMP-9, TIMP-2, and MMP-9/TIMP-1, while BDNF was significantly reduced(all p < 0.05). Other biomarkers, including IL 10, MDA, SOD, CAT, MMP-2, TIMP-1, β-NGF,and α2M, did not show significant within-pair differences.

**Table 1:** Clinical characteristics of monozygotic twins discordant for painful TMD based on BPI and DC/TMD. Δ values represent within-pair differences (TMD – Control)

Twin pair	Δ Pain severity (BPI)	Δ Pain interference (BPI)	Δ Reported pain points (%)	Δ Movement-evoked pain (%)	Δ Familiar pain on palpation (%)	Δ Referred pain on palpation (%)	Δ Headache on palpation (%)
Pair 1	–1.5	+ 3.5	0	0	+ 81	+ 62	+ 83
Pair 2	+ 3.8	+ 3.3	+ 7	+ 16	+ 44	+ 31	+ 16
Pair 3	+ 4.3	+ 1.1	+ 22	+ 8	+ 62	+ 56	+ 33
Pair 4	+ 0.5	+ 1.1	+ 21	+ 14	+ 19	+ 50	–17
Pair 5	+ 3.7	–0.3	0	+ 4	+ 6	+ 19	0
Pair 6	+ 1.7	+ 1.2	+ 29	+ 8	+ 100	+ 100	+ 100
Pair 7	+ 1.5	+ 1.8	+ 36	+ 4	+ 62	+ 12	+ 83
Pair 8	+ 5.3	+ 1.9	+ 28	0	+ 31	+ 12	+ 83
Pair 9	–2.0	–2.5	–14	–10	+ 6	+ 19	+ 50
Pair 10	–2.5	+ 3.3	+ 21	–4	–7	+ 19	–17
**Mean Δ ± SD**	**+ 1.48 ± 2.55**	**+ 1.44 ± 2.05**	**+ 15.0 ± 13.4**	**+ 4.0 ± 7.1**	**+ 40.4 ± 33.4**	**+ 38.0 ± 29.6**	**+ 41.4 ± 38.3**

Notes: Pain outcomes are expressed as percentages of positive responses relative to the number of sites evaluated: 14 sites for reported pain points (DC/TMD E1a–E1b), 50 sites for movement-evoked pain (DC/TMD E4b–E5c), 16 sites for familiar and referred pain on palpation (DC/TMD E9), 6 sites for headache-related palpation pain (temporal muscle; DC/TMD E9). All control twins fulfilled the DC/TMD “Absence of TMD” classification, although some exhibited subclinical findings (e.g., mild tenderness without diagnostic significance)

**Fig. 2:**
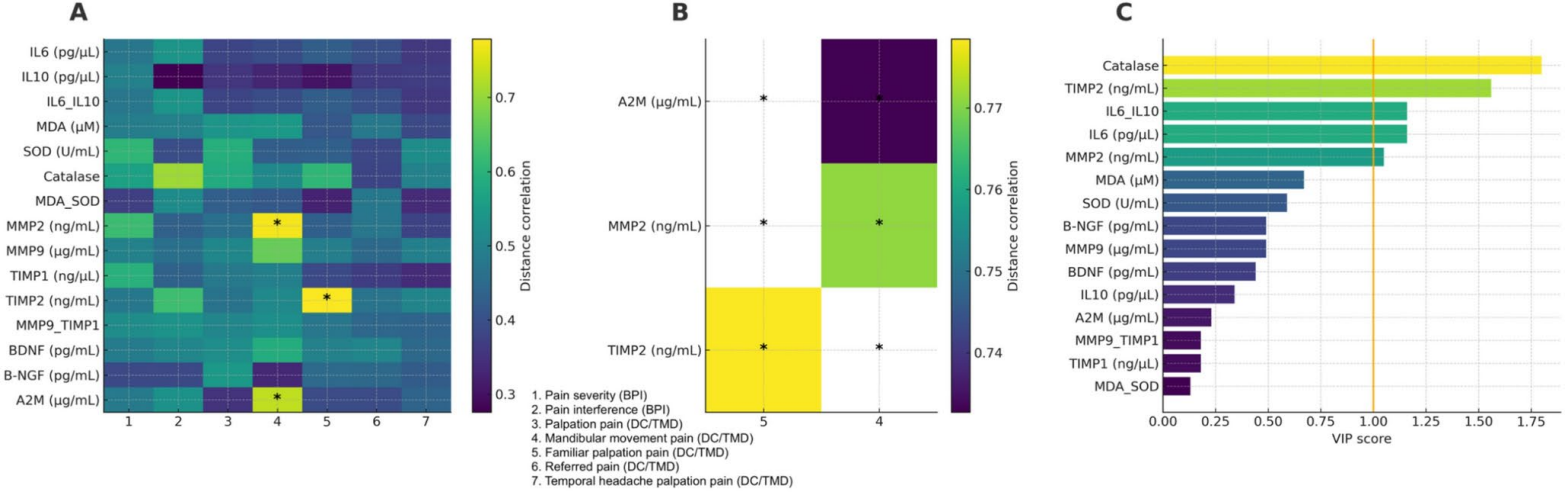
Multimodal association analyses between biomarker differences and clinical pain measures in monozygotic twins discordant for painful TMD. (**A**) Distance correlation matrix between biomarker deltas (Δ = TMD – Control) and clinical variables. Clinical variables: (1) Pain severity (BPI); (2) Pain interference (BPI); (3) Palpation pain (DC/TMD); (4) Mandibular movement pain (DC/TMD); (5) Familiar palpation pain (DC/TMD); (6) Referred pain (DC/TMD); (7) Temporal headache palpation pain (DC/TMD). A permutation-based distance correlation test (10,000 permutations) was used to capture linear and non-linear associations. Asterisks indicate statistically significant associations (p < 0.05). Main findings: IL-6, IL-6/IL-10, MDA, and MDA/SOD showed positive associations with multiple dimensions of pain, whereas BDNF and β-NGF displayed negative associations, indicating reduced neurotrophic support in individuals reporting higher pain levels. (**B**) Barplots of significant biomarker–clinical associations identified through distance correlation. Bars represent the magnitude of distance correlation for biomarker–clinical variable pairs showing p < 0.05 in panel A. Main findings: Inflammatory and oxidative markers (IL-6, IL-6/IL-10, MDA/SOD) and the matrix remodeling ratio MMP-9/TIMP-1 were the most consistent molecular correlates of pain severity, palpation tenderness, and movement-evoked pain. (**C**) Partial Least Squares (PLS) regression identifying biomarkers contributing most strongly to global clinical pain expression. PLS was performed using all biomarker deltas as predictors and all clinical variables as multivariate outcomes. Biomarkers with Variable Importance in Projection (VIP) > 1 were considered relevant contributors. Main findings: The PLS model highlighted an integrated inflammatory–oxidative axis (IL-6, MDA/SOD, MMP-9/TIMP-1) as the dominant molecular signature underlying the painful phenotype, while neurotrophic markers (BDNF, β-NGF) contributed inversely to overall pain burden.

The correlation analysis revealed coherent biological patterns linking biomarker shifts to clinical pain expression in monozygotic twins discordant for painful TMD (Fig. 2). IL-6, the IL-6/IL-10 ratio, MDA, and the MDA/SOD ratio—showed moderate to strong positive correlations with pain severity and pain interference on the BPI. In contrast, BDNF and β-NGF correlated negatively with BPI scores, suggesting that reduced neurotrophic support may be associated with more severe and disabling pain symptoms.

Associations between biomarker deltas and DC/TMD clinical variables followed a similar patterned structure (Fig. 2). A higher proportion of painful palpation sites and greater movement-evoked pain showed positive correlations with inflammatory and oxidative markers, mirroring the pattern observed for BPI scores. The MMP-9/TIMP-1 ratio was positively correlated with palpation pain and movement-evoked pain, suggesting that local tissue remodeling may accompany or reflect the mechanical sensitivity observed in the painful twins.

Taken together, these results demonstrate that the significant associations—highlighted by asterisks in Fig. 2—are concentrated within specific biological clusters rather than evenly distributed across all measured markers. Inflammatory, oxidative, and matrix-remodeling biomarkers consistently tracked with greater pain intensity, functional interference, and palpation-evoked pain, whereas neurotrophic markers showed the opposite trend. This selective correlation pattern supports the interpretation that painful TMD in genetically identical individuals aligns with a coordinated peripheral biological profile involving inflammation, oxidative imbalance, and increased matrix turnover.

**Fig. 3:**
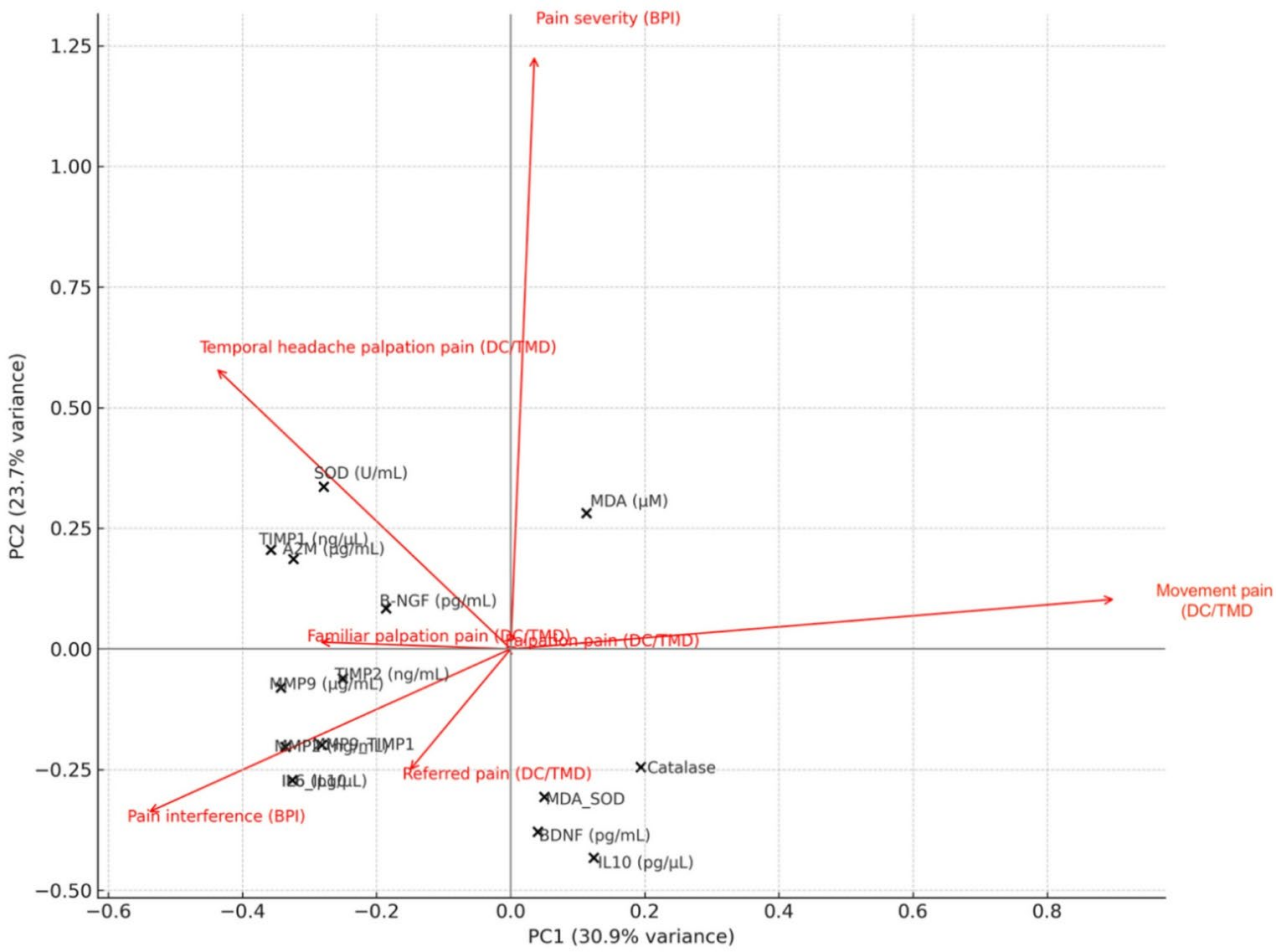
Principal component analysis (PCA) of within-pair biomarker differences (Δ = TMD – Control) with projected clinical vectors. Principal component analysis was performed on z-standardized within-pair biomarker deltas to explore multivariate covariation across inflammatory, oxidative stress, matrix-remodeling, and neurotrophic domains. Clinical variables were projected as supplementary correlation vectors to indicate their contribution to the latent components. PC1 and PC2 together captured the dominant multivariate structure of the dataset, with PC1 primarily reflecting a coordinated inflammatory–oxidative axis, while PC2 showed contributions from neurotrophic and matrix-remodeling factors. Longer vectors indicate stronger correlations between clinical outcomes and the biomarker-derived dimensions. Pain severity (BPI severity), pain interference (BPI interference), proportion of painful palpation sites (Spontaneous pain points, DC/TMD), movement-evoked pain (Movement pain, DC/TMD), and headache-related palpation tenderness (Headache palpation, DC/TMD) aligned positively with PC1, suggesting that greater clinical symptom burden was associated with higher pro-inflammatory, oxidative, and matrix-remodeling biomarker shifts. In contrast, BDNF and β-NGF loaded oppositely to these clinical vectors, consistent with a relative reduction in neurotrophic support among the more symptomatic twins. Collectively, the PCA reveals a coherent multidimensional biological signature linking peripheral molecular alterations to clinical pain expression in monozygotic twins discordant for painful TMD.

The multivariate structure of within-pair biomarker differences (Δ = TMD – Control) was further explored using PCA (Fig.  3). PC1 captured the predominant inflammatory–oxidative dimension of variation, with higher loadings for IL-6, the IL-6/IL-10 ratio, MDA, MDA/SOD, and MMP-9/TIMP-1. PC2 reflected secondary contributions from neurotrophic and matrix-remodeling markers. When projected as supplementary variables, clinical vectors—including pain severity and interference (BPI), palpation pain, movement-evoked pain, and headache-related palpation tenderness (DC/TMD)—aligned with the positive direction of PC1, suggesting that greater clinical symptom burden co-occurred with a biological shift toward heightened inflammatory, oxidative, and matrix-degrading profiles. Conversely, BDNF and β-NGF loaded in the opposite direction, consistent with their negative associations with clinical pain outcomes. In summary, PC1 represents a dominant inflammatory–oxidative dimension associated with greater clinical pain burden, whereas PC2 reflects secondary contributions from neurotrophic and matrix-remodeling pathways.

## Discussion

This study in monozygotic twins discordant for painful TMD demonstrated that twins with pain exhibited higher pain intensity, greater functional interference, and more widespread mechanical hyperalgesia, consistent with the multidimensional clinical features of myogenous TMD. These clinical differences were accompanied by selective alterations across inflammatory, oxidative, matrix-remodeling, and neurotrophic pathways. Rather than indicating global biological divergence, the findings revealed a coherent subset of biomarkers that differentiated painful twins from their co-twins, including higher IL-6 levels, an increased IL-6/IL-10 ratio, elevated MDA/SOD and MMP-9/TIMP-1 ratios, and lower BDNF. Although these changes were not uniform across all biomarkers, the overall pattern supports models in which the painful phenotype may reflect experience-dependent biological plasticity rather than fixed genetic predisposition1-3,6. These results align with contemporary pain neuroscience frameworks highlighting the interplay among immune dysregulation, oxidative imbalance, and neuroplastic adaptations in persistent pain [21–24].

A central strength of this study is the discordant monozygotic twin design, which provides a highly controlled framework to distinguish environmentally shaped biological processes from those related to heritable liability. Prior research indicates that TMD is moderately heritable but strongly influenced by environmental and behavioral factors [4, 7–9]. The present findings build on earlier work in this cohort demonstrating cognitive-emotional and somatosensory divergence between twins, extending this pattern to peripheral biological measures. Together, these observations suggest that interactions among stress exposure, maladaptive coping, and neuroimmune mechanisms may be involved in pain persistence, even when genetic factors are fully shared [2, 5, 6, 21, 22].

The most consistent inflammatory finding was the elevation of IL-6 and the IL-6/IL-10 ratio in the painful twins. This pattern is compatible with evidence supporting low-grade inflammatory activation in chronic TMD and related musculoskeletal pain conditions [1, 3, 6, 14]. IL-6 has well-established roles in nociceptor sensitization and neuroimmune amplification [10, 12, 13, 22]. The stability of IL-10 across twins may suggest limited compensatory anti-inflammatory buffering, echoing literature proposing that chronic pain reflects an imbalance between pro- and anti-inflammatory mediators.^3^,^15^,^21^,^24^ Although IL-6 and the IL-6/IL-10 ratio were elevated in painful twins, these findings should not be interpreted as evidence of a generalized inflammatory state. Rather, they indicate a pro-inflammatory profile that, in combination with oxidative and matrix-remodeling markers, suggests coordinated peripheral biological alterations associated with painful TMD. Although the biomarker panel captured multiple interacting biological pathways, it did not include a comprehensive set of pro-inflammatory cytokines such as TNF-α or IL-1β. Future studies incorporating a broader inflammatory profile may further refine the characterization of these biological patterns.

Higher MDA/SOD among painful twins, combined with alterations in antioxidant enzyme activity, is compatible with mechanisms involving lipid peroxidation, mitochondrial stress, and amplified nociceptive signaling [1, 3, 6, 23]. Oxidative pathways frequently act in concert with inflammatory mediators to sustain neuroimmune activation and hinder resolution of inflammation [12–14, 25]. The growing body of evidence implicating redox mechanisms in TMD pain enhances the translational relevance of these observations [3, 15, 23, 24].

Altered extracellular matrix remodeling, reflected in higher MMP-9 and an increased MMP-9/TIMP-1 ratio, may be associated with peripheral sensitization. MMP-9 is increasingly recognized as a contributor to neuroinflammation, nociceptor plasticity, and hyperalgesia [12, 21, 22, 25]. Although not all remodeling markers differed significantly, the pattern observed aligns with models postulating that matrix turnover may accompany or amplify local tissue reactivity in myogenous TMD [10, 12, 14, 15].

In contrast, neurotrophic markers showed decreased BDNF and β-NGF in painful twins. Although neurotrophins can contribute to nociceptor sensitization, they also support neuronal repair and adaptive plasticity [3, 10, 11, 16]. Lower levels in the painful twins may be linked to chronic stress exposure, reduced homeostatic capacity, or environmentally shaped epigenetic changes [21, 26–28]. These results are compatible with evidence that monozygotic twins can diverge epigenetically over time through differential life experiences and stress load [11, 16, 28, 29].

The multivariate analyses further supported these domain-specific findings. PCA identified a dominant inflammatory-oxidative component that aligned with clinical pain vectors, alongside a secondary anti-inflammatory/antioxidant axis moving in the opposite direction [1, 5, 10, 22]. This composite structure is consistent with integrated pain models situating chronic pain within dynamic interactions across immune, metabolic, and neural systems [3, 21, 23, 24].

This study has limitations, including the modest sample size inherent to discordant twin designs and its cross-sectional nature, which precludes causal inference. Central nervous system measures, such as neuroimaging or assessments of descending pain modulation, would further enhance the interpretation of these peripheral findings [1, 3, 15, 30]. This framework is not achievable in studies of unrelated individuals and strengthens the interpretation that the observed inflammatory, oxidative, and neurotrophic patterns may reflect lived experience rather than inherited predisposition. In this context, the convergence of clinical and biomarker alterations across immune, redox, and neurotrophic pathways is consistent with models of biological embedding in chronic TMD pain [1, 21, 24, 29].

In summary, this genetically controlled study indicates that painful TMD in monozygotic twins is characterized by coordinated but pathway-specific alterations across inflammatory, oxidative, remodeling, and neurotrophic domains. These findings reinforce conceptual models in which chronic orofacial pain emerges through environmentally shaped biological processes. A deeper understanding of these pathways may support mechanistically informed and individualized phenotyping in TMD [5, 14, 15, 24]. These biomarker profiles may contribute to clinical stratification by identifying subgroups of patients with distinct inflammatory–oxidative and neurotrophic patterns. Such differentiation could support the development of more personalized therapeutic approaches, targeting specific biological pathways involved in painful TMD.

In conclusion, in monozygotic twins discordant for painful TMD, the painful twin exhibited greater clinical burden along with selective peripheral biological alterations, including increased inflammatory and oxidative activity, elevated matrix-remodeling markers, and reduced neurotrophic support. These coordinated, pathway-specific differences emerged despite genetic identity, may reflect that chronic TMD pain reflects environmentally and experientially shaped biological changes. Collectively, these findings support peripheral molecular pathways as potential targets for mechanistically informed and personalized approaches to pain-related TMD.

## Data Availability

Data will be made available upon request to the corresponding authors.
